# The Healthy Pregnancy Research Program: transforming pregnancy research through a ResearchKit app

**DOI:** 10.1038/s41746-018-0052-2

**Published:** 2018-09-05

**Authors:** Jennifer M. Radin, Steven R. Steinhubl, Andrew I. Su, Hansa Bhargava, Benjamin Greenberg, Brian M. Bot, Megan Doerr, Eric J. Topol

**Affiliations:** 1 Digital Medicine Division, Scripps Research Translational Institute, La Jolla, CA USA; 2 Wave Research Center, La Jolla, CA USA; 3 WebMD, New York, NY USA; 4 Sage Bionetworks, Seattle, WA USA

**Keywords:** Epidemiology, Risk factors, Diseases, Reproductive signs and symptoms

## Abstract

Although maternal morbidity and mortality in the US is among the worst of developed countries, pregnant women have been under-represented in research studies, resulting in deficiencies in evidence-based guidance for treatment. There are over two billion smartphone users worldwide, enabling researchers to easily and cheaply conduct extremely large-scale research studies through smartphone apps, especially among pregnant women in whom app use is exceptionally high, predominantly as an information conduit. We developed the first pregnancy research app that is embedded within an existing, popular pregnancy app for self-management and education of expectant mothers. Through the large-scale and simplified collection of survey and sensor generated data via the app, we aim to improve our understanding of factors that promote a healthy pregnancy for both the mother and developing fetus. From the launch of this cohort study on 16 March 2017 through 17 December 2017, we have enrolled 2058 pregnant women from all 50 states. Our study population is diverse geographically and demographically, and fairly representative of US population averages. We have collected 14,045 individual surveys and 107,102 total daily measurements of sleep, activity, blood pressure, and heart rate during this time. On average, women stayed engaged in the study for 59 days and 45 percent who reached their due date filled out the final outcome survey. During the first 9 months, we demonstrated the potential for a smartphone-based research platform to capture an ever-expanding array of longitudinal, objective, and subjective participant-generated data from a continuously growing and diverse population of pregnant women.

## Introduction

Historically, pregnant women, and even women of reproductive potential, have been under-represented in research, due to potential harm to the fetus.^1^ This lack of research has led to gaps in evidence-based treatment, interventions and guidelines for women,^2^ especially ones that are individualized to reflect diverse characteristics of all pregnant women.^1,3^ Since nearly four million women in the US give birth every year,^4^ pregnancy is a topic that deserves far greater attention and research focus.

Given the growing population of mobile internet users, which is expected to reach 60% of the global population by 2020,^5^ we have a unique opportunity to access and enroll a diverse and large population of pregnant women across the US. There are also a growing array of digitally connected devices and sensors, which will enable us to quantify and collect more frequently and accurately measured metrics from the expectant mother’s real world than ever before, allowing for a uniquely detailed understanding of individual variations in physiologic changes during pregnancy. In order to explore this potential, we developed a smartphone app research platform called the Healthy Pregnancy Research Program. The overall purpose of this Program is to develop an ever-improving, user-centric platform that will serve as a global resource for the collection of a wide range of valuable longitudinal health data throughout pregnancy, and then use that data to increase the knowledge of the individual user as well as the research community to help identify the characteristics that create the healthiest pregnancy for an individual. The current report describes the Healthy Pregnancy app’s early effectiveness and important learnings in enrolling and engaging a large and diverse population of women throughout the duration of their pregnancy, and highlights the ability to collect detailed and useful objective and subjective data.

## Results

### Recruitment and enrollment

From the study’s launch on 16 March 2017 through 17 December 2017, there were 49,411 unique views of the welcome screen. Out of these, 9438 self-identified that they met the inclusion criteria for the study.

**Fig. 1:**
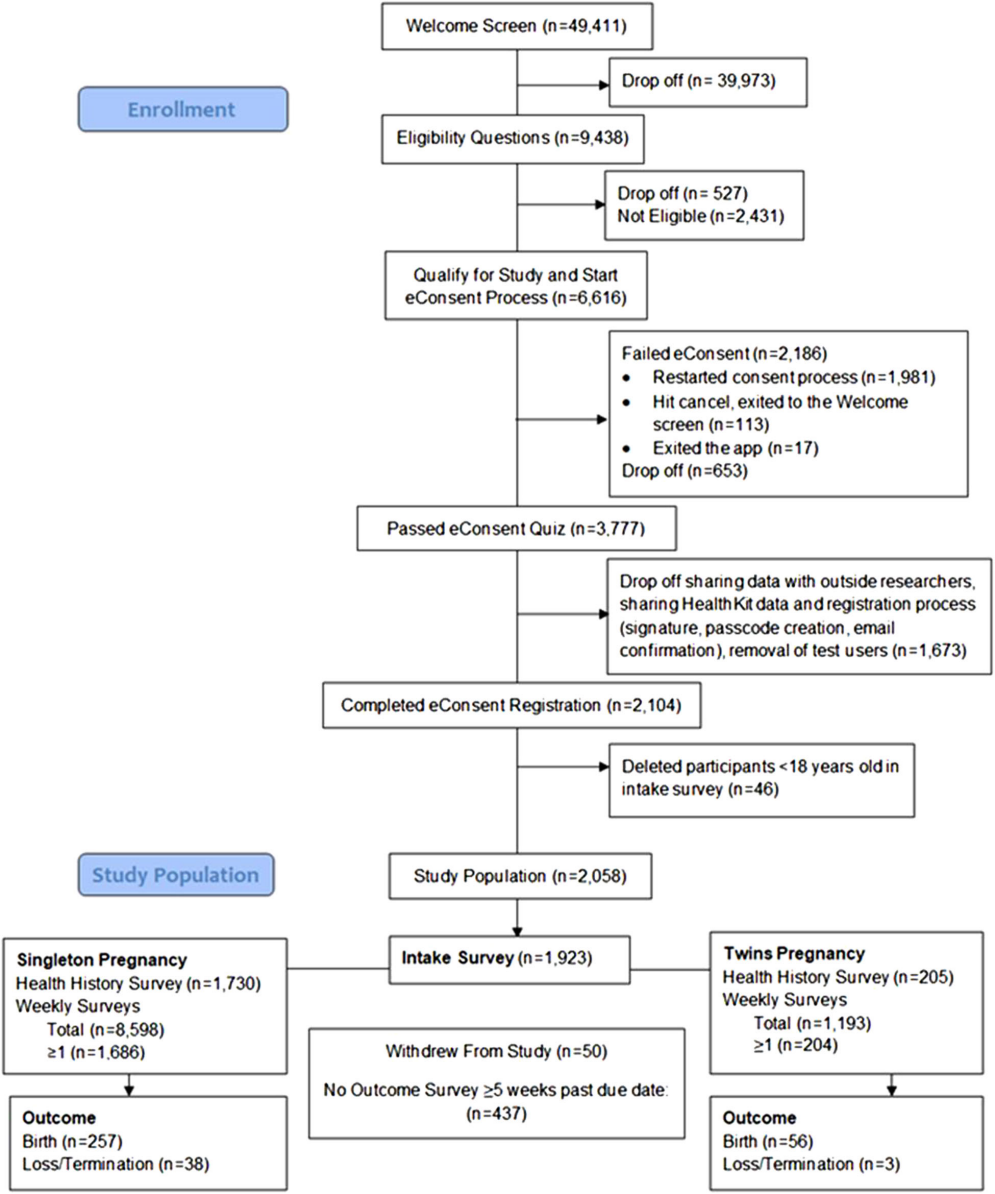
Consort diagram of participant enrollment, 16 March 2016–17 December 2017. Participants can choose to fill out the intake survey, health history survey, and weekly survey in any order

As participants read through the electronic Consent (eConsent) screens, we observed a gradual drop-off in the number of unique screen views. Additionally, very few people opted to read the long, detailed “learn more” version of each topic (0.4–3.8%). Interestingly, certain topics such as “Potential Risks,” and “Future Independent Research” got higher number of views for the detailed “learn more” screens (Supplementary Fig. 1). The first comprehension question of the eConsent quiz was viewed by 6558 unique users. A total of 2186 users failed the quiz and in the end, a total of 3777 (58% of those who started quiz) made it to the “comprehension passed” screen. However, it is unclear how many of these unique users were simply testing out the app and the eConsent process, instead of real potential users. Out of these, an additional 1673 dropped off during the screens that gave users the option to share HealthKit data, share data with outside researchers and during the registration process that included passcode creation and email confirmation. We also filtered out users who put “Test” as their username or were clearly test users based on their name. After removing 46 individuals who were less than 18 years old in the short intake survey, we were left with a total of 2058 participants in our study population (Fig. 1).

### Sample size and diversity

Currently, we have enrolled 2058 participants from all U.S. states, Puerto Rico and the US Virgin Islands, with the highest number of enrollees from highly populated states such as California, Texas, and New York. (Supplementary Fig. 2) Fifteen percent of our population comes from rural zip codes (zip codes without a Metropolitan Statistical Area code) compared to 19% in the entire US population.^6^

We enrolled more women in the 30–39 year age group and less in the 20–29 year age group compared to national averages for pregnant women.^4^ The percentage of women in each body mass index (BMI) category was similar to national averages for pre-pregnancy weights^7^ (Table 1 and Supplementary Fig. 3). Although we have enrolled fewer participants from some racial minorities compared to US averages, the percentage of non-White participants was very close to national averages (Table 1).

**Table 1:** Healthy pregnancy population characteristics 16 March 2017–17 December 2017 and US maternal averages

Characteristic	Data missing (*n*)	*n* (%)	US average %
Short intake survey (*n* = 1923)			
Age (years), Mean (SD)	35	30.4 ± 5.8	26.4^a,^^4^
Age category (years)			
18–19		75 (4.0)	4.3
20–29		788 (41.7)	51.2
30–39		944 (50.0)	41.5
>40		81 (4.3)	3.1^4^
Race/ethnicity^b^	0		
White		1438 (74.8)	75.7
Black or African American		248 (12.9)	16.1
Hispanic or Latino		237 (12.3)	23.2
Asian		80 (4.2)	7.1^c^
Native Hawaiian or other Pacific Islander		12 (0.6)	
American Indian or Alaskan Native		48 (2.5)	1.1^4^
Middle Eastern or North African		16 (0.8)	
Other		38 (2.0)	
Live in rural zip code	76	280 (15.2)	19.3^6^
Pre-pregnancy weight, kgs, Mean (SD)	26	72.8 ± 19.7	
Pre-pregnancy BMI (kg/m^2^)	32		
Underweight (<18.5)		105 (5.6)	3.8
Normal (18.5–24.9)		850 (45.0)	45.9
Overweight (25.0–29.9)		475 (25.1)	25.6
Obese (≥30.0)		461 (24.4)	24.8^7^
Share data with all qualified researchers	0	1404 (73.0)	
Health history survey (*n* = 1935)			
Twin pregnancy		204 (10.8)^d^	3.3^4^
First pregnancy	13	629 (32.7)	
Prior miscarriage	23	627 (32.8)	16^15^
Pre-existing conditions			
Anxiety and/or depression	11	352 (18.3)	18.1^e,^^20^
Hypertension	0	66 (3.4)	6.8–19.0^f,^ ^21^
Eating disorder	0	50 (2.6)	0.3–3.5^g,^^22^

*SD* Standard deviation, *BMI* body mass index

^a^Age at first pregnancy, 2014

^b^Women may identify more than one race/ethnicity

^c^Asian and Pacific Islander

^d^Based on number of participants who filled out health history surveys

^e^Percentage is for entire US population over 18 years old

^f^Percentage of US women aged 20–34 years old, and 35–44 years old, respectively, in 2015

^g^Lifetime prevalence for US women, anorexia nervosa, (0.3), bulimia nervosa (1.5), and binge eating disorder (3.5), 2001–2003

### Engagement

We found that our study population was engaged by the study surveys, with 93.4% of enrollees filling out the intake survey, 94.0% filling out the health history survey, 91.8% filling out at least one weekly survey, and 84.3% of participants opting to share HealthKit data with the study. Currently, we have collected 109,047 total daily HealthKit measurements, with 81,673 days of step measurements, 11,046 days of sleep measurements, 13,842 days of heart rate (HR) measurements, 1945 days of weight measurements, and 541 days of BP measurements (Supplementary Table 1). Over the course of a single day, there were often tens to thousands of HR, distance and step count measurements from participants wearing a connected device such as an Apple Watch.

Among participants who reached their due dates, we found that participants with singleton pregnancies filled out an average of 6 surveys over 56 days (74,200 total person-days of follow-up) and women with twin pregnancies filled out an average of 7 surveys over 82 days (13,202 total person-days of follow-up). Forty-five percent of women completed their final outcome survey, out of the 791 women who went to full term or had an earlier outcome (Fig. 1).

### App collected data

**Fig. 2:**
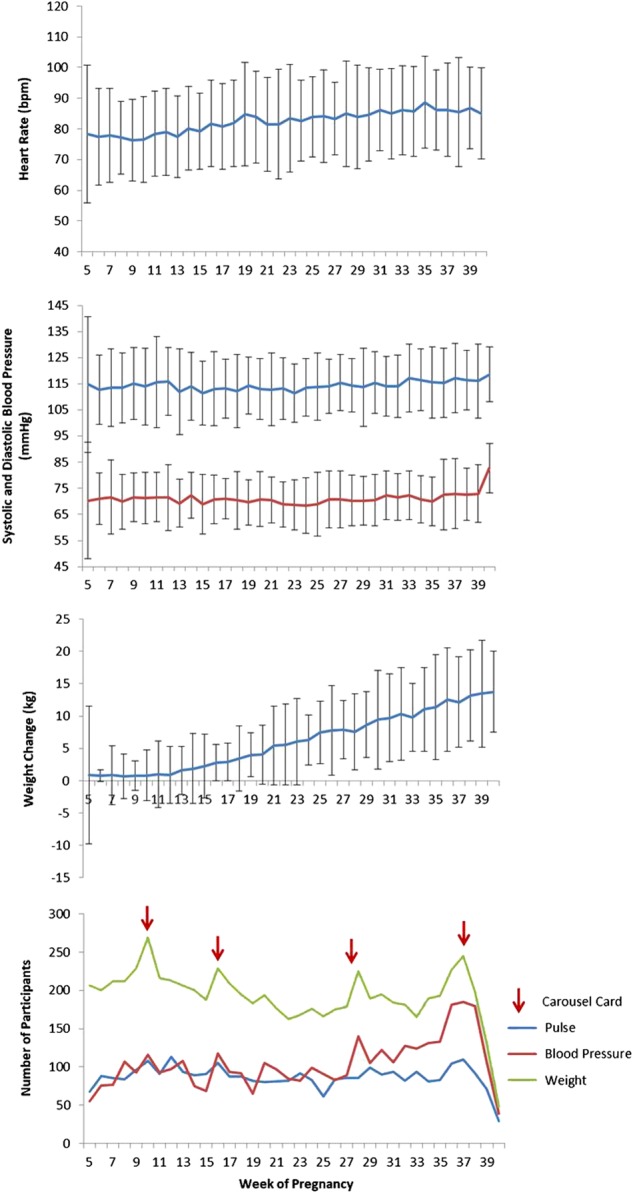
Mean (SD) physiological changes by pregnancy week from self-reported data singleton pregnancies, 16 March 2016 to 18 December 2017. Top to bottom: heart rate, systolic (blue) and diastolic (red) blood pressure, and weight change from pre-pregnancy weight, and number of participants sharing data each week

**Fig. 3:**
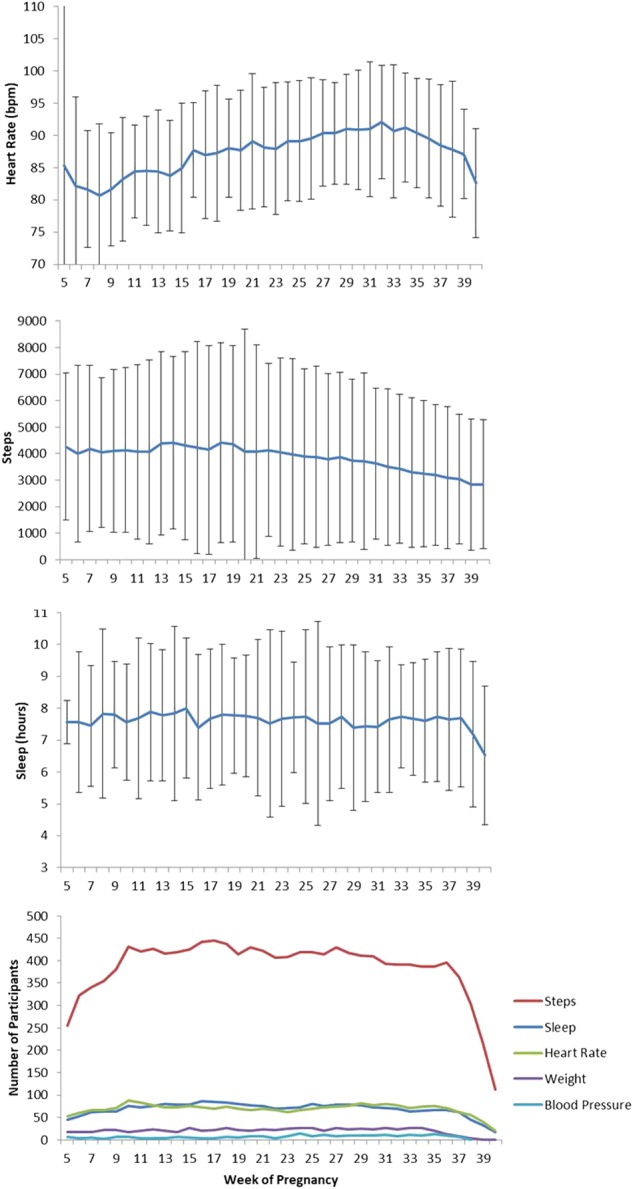
Mean (SD) of participant’s daily HealthKit data by pregnancy week, 16 March to 18 December, 2017. Top to bottom: heart rate, steps, sleep, and number of participants sharing data each week

During the course of pregnancy, population averages of self-reported and HealthKit collected HR rose. However, there was a declining trend for HR in the HealthKit data near the end of pregnancy, which may be a result of some women delivering their baby’s early and not filling out a final outcome survey while their HealthKit data was still being collected. On average women reported gaining around 13 kilograms by the end of their pregnancy, with a slower rate of weight gain during the first trimester. Total weekly step count appeared to decline steadily during the late second and third trimester. Average sleep remained relatively stable around 7.5 h per night. A larger population size will enable us to evaluate some of these trends closer by individual characteristics. (Figs. 2 and 3).

We saw increases in participants joining and self-reporting measurements during their weekly questionnaire around the time when the overall WebMD pregnancy app had carousel cards promoting the study (week 10, 16, 27, and 36) (Fig. 2 and Supplementary Figs. 4 and 5). When looking at the number of weekly surveys filled out with weight measurements, we found that women who joined around week 4–5, and 16–17, and during the last trimester were more likely to fill out surveys consistently over time and also filled out more outcome surveys (Supplementary Fig. 4)

The top ten most prescribed medications from the health history survey included drugs to treat or prevent anti-depression, thyroid deficiency, miscarriage, morning sickness, and diabetes. The top ten most taken over the counter drugs were prenatal vitamins, and drugs for pain relief, allergy, nausea and heartburn, sleep, and probiotics. Although, the FDA is moving away from letter risk categorization of medications, 6 of the top 10 prescribed medications and 2 of the top 10 over-the-counter drugs were category C, meaning risk is not ruled out and animal reproduction studies have shown possible adverse effects in the fetus (Table 2).

**Table 2:** List of the top ten most prescribed and over-the-counter medications during pregnancy, drug indication, Food and Drug Administration pregnancy category, and percentage of participants taking the drug

Prescribed medications	Over-the-counter
1. Antidepressants (8%)	1. Prenatal vitamins (93%)
a. Zoloft, C (4%)	2. Analgesic (9%)
b. Bupropion, C (1%)	a. Acetaminophen, B (5%)
c. Prozac, C (1%)	b. Aspirin (NSAID), C/D (4%)
d. Celexa, C (1%)	3. Allergy (7%)
e. Lexapro, C (1%)	a. Zyrtec, B (3%)
2. Levothyroxine (thyroid deficiency), C (5%)	b. Claritin, B (2%)
3. Progesterone (Infertility/prevent miscarriage), A (2%)	c. Benadryl, B (2%)
4. Morning sickness (3%)	4. Zantac (nausea/heartburn), B (2%)
a. Diclegis, A (2%)	5. Unisom (sleep), B (2%)
b. Zofran, B (1%)	6. Probiotic (healthy gut), not assigned (2%)
5. Metformin (type 2 diabetes), B (1%)	7. Tums (antacid), C (1%)

Data is from the health history survey for singleton pregnancies, 16 March–17 December, 2017, *n* = 1730

## Discussion

During the first nine months of the deployment of the Healthy Pregnancy ResearchKit app, we have enrolled over 2000 participants from 50 states, and collected over 14,000 individual surveys and 100,000 daily HealthKit measurements. Going forward, as we incorporate a greater number of enrollees, we plan to incorporate more novel digital devices for an even greater variety and volume of participant-generated data. Overall, we believe this app can prove to be on ongoing, ever-improving source of important insights to better understand the individual factors that create a healthy pregnancy for all women.

Over the last decade there has been a shift in how pregnant women find and share pregnancy related health information. A recent study found through a survey of pregnant women that 55% were using an app related to pregnancy, birth, and/or child care.^8^ In fact, 7% of all mHealth apps in 2015 were focused on women’s health and pregnancy.^9^ These apps provide useful tailored information based on week of pregnancy at their fingertips. High pregnancy app usage combined with an increasing number of people using digitally connected devices and sensors to monitor their health, allows for unique possibilities to collect and provide information that is tailored to individual consumers. These technologies also have the ability to transform research by enabling the sharing of participant-generated data in a way that is less burdensome to participants.

**Fig. 4:**
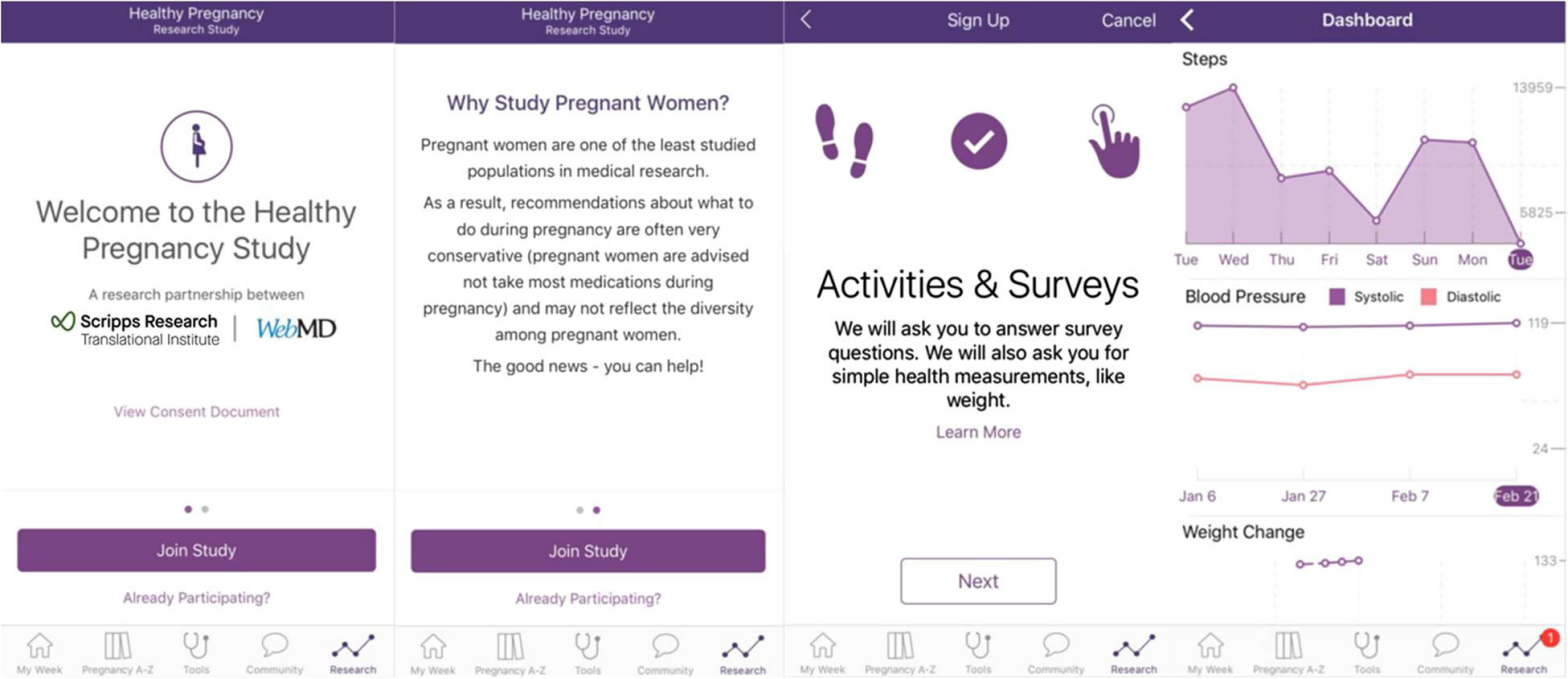
Screenshots of the Healthy Pregnancy Study. Left to right: (1) Welcome screen (2) Why study pregnant women? (3) Activities and surveys (4) Dashboard tracking physiological and activity changes during pregnancy

One unique aspect of the Healthy Pregnancy Research Program is that it is embedded in WebMD’s highly trafficked pregnancy app (Fig. 4). Since 2013, over 1.6 million people have downloaded WebMD’s Pregnancy App. WebMD’s Pregnancy App is medically reviewed by physicians and provides answers to many questions that women would typically ask their health care provider. We believe that by incorporating our study platform into a trusted app where pregnant women are already going for their information has given us high visibility and likely better long-term engagement. Efforts are underway to embed our app into other trusted digital platforms.

We found that participants were more likely to read the detailed version of specific eConsent topics (e.g., Your Privacy, Potential Risks, and Future Independent Research), which may indicate additional interest, confusion, or concern for these topics. Currently we observed substantial drop-offs throughout the eConsent process, with 33% of participants failing the short eConsent quiz. In the future, giving participants who failed an eConsent question a description of why their answer was incorrect, instead of forcing them to re-do the entire eConsent process, in other words, restructuring the quiz from a summative to a formative evaluation, may improve enrollment. This teaching/reinforcing approach may also help improve participant understanding within the consent process. Additionally, identifying which questions were answered incorrectly is important for improving our understanding of where we are failing to adequately explain topics to participants and perhaps explore additional formats for eConsent presentation, such as voiceover or video. We plan to conduct qualitative and quantitative surveys of both current and potential participants to better understand what motivates participation and retention and how to improve the design, usability, and return of information within the app. In particular, we plan to better understand health literacy, acceptability of survey questions and sensors, and the overall needs of high risk and understudied populations (African American women, low income, and rural populations) who are likely to benefit the most from this research.

Relative to other ResearchKit studies, we have seen less attrition and a higher percentage or participants filling out the surveys.^10–13^ Additionally, 73% of participants in our study were willing to share data with outside researchers, which is similar to other ResearchKit studies (67–78%).^10,12,13^ Another pregnancy cohort study that approached women during clinic visit, enrolled a similar number of participants during a period that was more than three times as long as our study.^14^ Higher engagement and willingness to share data may be a result of our unique study population of expectant mothers bringing their baby into the world, during a limited, circumscribed temporal window. We may also have lower drop-off because women receive other useful information through the WebMD pregnancy app and may, therefore, be less likely to delete it and withdraw from the study. In order to improve long-term engagement, it will be necessary to continuously refine the return of useful information to participants. As more data is gathered from a greater number of women it will become possible to eventually match a woman with their “digital twin” to help more precisely guide their expectations and health behaviors.

Certain participant characteristics seem to influence their likelihood of participating. Interestingly, 3% of all births in the US are twin births;^4^ however, our study is capturing a much larger proportion of twin pregnancies (11%). Similarly, we saw a higher percentage of women joining our study with prior miscarriages (33%) compared to national averages (10–20%)^15^ (Table 1). Women also joined our study on average at gestational week 17. Finding ways to encourage women to join earlier will allow us to collect important data that may help us identify signs of early complications. Overall, higher engagement among certain sub-populations or during certain times of pregnancy may be a result of heightened concern or increased questions during these times or for these groups. These groups may be more motivated to be part of a research study with the hope of answering their questions or improving their particular condition.

Traditional clinic-based studies have often lacked participant diversity due to participation challenges such as lack of access to care centers that typically refer people to research studies, time constraints for participation, difficulties with transportation to research sites, lack of child care while participating, and limited participation hours.^16^ One pregnancy cohort study found lower long-term engagement among women who were younger, non-White, and who had lower income levels, lower education, and more likely to be un-married and suffer from poor psychosocial health.^17^ Additionally, traditional cohort studies are often limited to one or only several geographic locations, whereas we have easily enrolled participants from across the US. By conducting an app-based research study that takes limited time, and can be done anywhere and at any time, we have helped overcome some of these challenges, which has likely increased our overall participant diversity. However, we believe that through continued refinement of the recruitment strategies, app design, usability, and return of information, we will be able to further improve the apps penetration to high risk and under-represented groups. One current limitation is our study is only available in iOS and iPhones are still relatively expensive, therefore, potentially biasing our study population to higher-income individuals (socioeconomic data was not collected). Future expansion to Android phones and HTML is also essential and will enable us to access a much wider and varied user population in the US and eventually, globally.

As the availability of an increasing variety of wireless, connected sensors grows, we anticipate including the automated daily (or even more frequent) collection of multiple parameters known to be germane to pregnancy such as BP, HR, activity, sleep, stress, nutrition, and glucose levels. We can also assess the impact of new digital platforms and home-based sensors at improving positive behavior change to improve health. In addition to conducting research, a primary future objective of this study is to help women meaningfully interpret and understand their personal data through visualizations, risk profiles, and comparisons to other individuals like them. Ultimately, this will make for more informed decisions for pregnant women when it comes to things from medication choices, to healthy weight gain and ideal sleep during pregnancy. Additionally, participant collected data from this app, such as BP changes over time, can potentially be shared with health care providers to help identify pregnancy complications and better understand an individual’s “normal” values. Future evaluation of how to best aggregate and share this data with clinicians in a manner that is useful and not burdensome is also necessary.

In summary, we aim to use our app-based research platform to help fill in many knowledge gaps that exist for pregnancy, ranging from individualized weight gain recommendations to earlier identification of preeclampsia, gestational diabetes, peripartum depression, and other complications. The first nine months of our study have proven the app’s success to quickly and easily collect detailed and useful data remotely from a large and relatively diverse population from across the US. These data may inform both improved population-based public health interventions and individualized care that will ultimately help create healthier pregnancies.

## Methods

### Overview and goals of the research program

The Healthy Pregnancy Research Program is a ResearchKit app-mediated study developed by Scripps Research Translational Institute and WebMD. ResearchKit is an open-source framework for building research apps, created by Apple (Apple Inc. *ResearchKit*, http://researchkit.org/). It allows researchers to conduct studies quickly, cheaply, and easily by collecting survey and HealthKit data through a dedicated study app on the participant’s iPhone (Apple Inc. *ResearchKit*, http://researchkit.org/).^18^ Our ResearchKit study can be accessed via the WebMD Pregnancy app (Fig. 4) or can be downloaded directly from the iTunes store.^19^

Data collected include maternal weight change, blood pressure (BP), medication usage, symptoms, diagnoses such as preeclampsia and gestational diabetes, as well as birth outcomes, primarily through participant-reported surveys. We are additionally collecting maternal activity data (steps and distance run/walked), sleep duration, weight, HR, and BP directly through the Apple iPhone’s HealthKit. The majority of HealthKit data is likely automatically uploaded from sensors; however, it is possible that some of the HealthKit data is also self-reported and manually entered. At this time, our app does not identify how the HealthKit data was collected. Currently, activity, weight and BP data is plotted in a graph so that participants can see how their individual data changes throughout their pregnancy. Participants also have the option to download their study data and have it emailed to them by clicking on a button in the app. This data can then be shared with their health care provider.

This study was approved by Scripps Research’s Institutional Review Board on 17 February 2017. The study was also registered on ClinicalTrials.gov (Identifier: NCT03085875) on 21 March 2017.

SAS version 9.4 was used for all analyses.

### Qualification and eConsent

Any pregnant person, 18 years or older, who lives in the US and is comfortable reading and writing on their iPhone in English is eligible to join our study. After self-identifying that they qualify for the study, potential participants self-navigate an eConsent that includes 17 screens highlighting key consent topics ranging from data sharing and data privacy, to potential risks. The consent process is entirely self-guided and self-administered, with no in-person steps. Within the eConsent process, participants can click on a “learn more” option to get a more detailed description of each topic or advance to the next screen. If a potential participant has questions regarding the eConsent, they are given the contact information of the study coordinator and Institutional Review Board. After completing the eConsent, participants must correctly answer four questions that test their comprehension of core study topics in order to join the study. Those who do not answer correctly are directed to the beginning of the eConsent process.

### Surveys

After completing the eConsent process, participants are given a short intake survey, health history survey and a weekly survey. The weekly survey recurs every week and initially asks participants if they are still pregnant, had a miscarriage or stillbirth, if the pregnancy ended, or if they gave birth. If they are still pregnant, the weekly survey asks recurring questions about physiological measurements, medications, and symptoms. If a participant had a prenatal visit during that week, they are given a few additional questions about vaccines and diagnoses. It is possible that data collected from the surveys on HR, BP, and weight overlaps with some of the HealthKit data. Although, participants can only submit one measurement a week via their weekly survey, they can automatically upload or manually add as many HealthKit data points as they desire. If they indicate a birth, they are given the outcome survey questions about the baby’s weight and length, and labor and delivery questions. An outcome survey is also given 4 weeks after a participant’s due date, if they do not indicate a birth outcome during the weekly survey (Supplementary Table 2).

At any time, participants are able to skip over questions that they do not want to answer. There are also some built in blocks that do not allow participants to fill in likely inaccurate values (for example: pregnant women’s weight over 500 pounds, baby’s weight over 18 lbs, HR over 220 beats per minute). For our HealthKit analysis, we deleted any daily step counts less than 500 per day, HRs less than 30 beats per minute, weekly average sleep less than 1 h, and BP measurements with systolic less than 70 mm Hg and diastolic less than 50 mm Hg. Our sleep data summed all of an individual’s sleep for 1 day, including possible naps. It is possible, that our app pulled HealthKit data after women gave birth if their baby arrived before their due date.

## Electronic supplementary material

Supplementary Information

## Data Availability

The datasets generated during and/or analyzed in this study are available from healthypregnancy@scripps.edu on reasonable request.

## Electronic supplementary material

**Supplementary information** accompanies the paper on the *npj Digital Medicine* website (10.1038/s41746-018-0052-2).

## References

[1] Baylis F (2010). Pregnant women deserve better. Nature.

[2] Kim AM, Tingen CM, Woodruff TK (2010). Sex bias in trials and treatment must end. Nature.

[3] Putting gender on the agenda. *Nature* **465**,665(2010). 10.1038/465665a 20535156

[4] Martin JA, Hamilton BE, Osterman MJ, Driscoll AK, Mathews TJ (2017). Births: Final data for 2015. Natl. Vital. Stat. Rep..

[5] GSMA Intelligence. *The Mobile Economy*, https://www.gsmaintelligence.com/research/download (2017).

[6] United States Census Bureau. *2010**Census Urban and Rural Classification and Urban Area Criteria*, https://www.census.gov/geo/reference/ua/urban-rural-2010.html (2010).

[7] Branum AM, Kirmeyer SE, Gregory EC (2016). Prepregnancy body mass index by maternal characteristics and state: Data from the birth certificate, 2014. Natl. Vital. Stat. Rep..

[8] Lee Y, Moon M (2016). Utilization and content evaluation of mobile applications for pregnancy, birth, and child care. Healthc. Informa Res..

[9] IMS Institute for Healthcare Informatics. *Patient Adoption of mHealth: Use, Evidence and Remaining Barriers to Mainstream Acceptance* http://static.correofarmaceutico.com/docs/2015/09/21/iihi_patient_adoption_of_mhealth.pdf (2015).

[10] Webster DE (2017). The Mole Mapper Study, mobile phone skin imaging and melanoma risk data collected using ResearchKit. Sci. Data.

[11] McConnell MV (2017). Feasibility of obtaining measures of lifestyle from a smartphone app: The MyHeart Counts Cardiovascular Health Study. JAMA Cardiol..

[12] Chan YY (2017). The Asthma Mobile Health Study, a large-scale clinical observational study using ResearchKit. Nat. Biotechnol..

[13] Bot BM (2016). The mPower study, Parkinson disease mobile data collected using ResearchKit. Sci. data.

[14] Oken E (2015). Cohort profile: project viva. Int. J. Epidemiol..

[15] Ventura SJ, Mosher WD, Curtin SC, Abma JC, Henshaw S (1999). Highlights of trends in pregnancies and pregnancy rates by outcome: estimates for the United States, 1976-96. Natl. Vital. Stat. Rep..

[16] Oh SS (2015). Diversity in clinical and biomedical research: A promise yet to be fulfilled. PLoS Med..

[17] McDonald SW (2013). The all our babies pregnancy cohort: design, methods, and participant characteristics. BMC Pregnancy Childbirth.

[18] Jardine J, Fisher J, Carrick B (2015). Apple’s ResearchKit: smart data collection for the smartphone era?. J. R. Soc. Med..

[19] iTunes. *WebMD Pregnancy*, https://itunes.apple.com/us/app/webmd-pregnancy/id600535431?mt=8.

[20] Anxiety and Depression Association of America. *Facts and Statistics*, https://adaa.org/about-adaa/press-room/facts-statistics.

[21] Centers for Disease Control and Prevention. *High Blood Pressure Facts*, https://www.cdc.gov/bloodpressure/facts.htm.

[22] Hudson JI, Hiripi E, Pope HG, Kessler RC (2007). The prevalence and correlates of eating disorders in the National Comorbidity Survey Replication. Biol. Psychiatry.

