# Supplementary material for: The Healthy Pregnancy Research Program: transforming pregnancy research through a ResearchKit app
Source: NPJ Digit Med. 2018 Sep 5;1:45. doi: 10.1038/s41746-018-0052-2 (PMC6550256; doi:10.1038/s41746-018-0052-2)

Supplementary Table 1. HealthKit data from March 16, 2017 to December 17, 2017

	Total Number of Participants Who Shared	Total Number Measurement Days	Total Number of Measurements	Average Number of Measurements Per Person
Steps	1,426	81,673	81,673*	57
Sleep	294	11,046	11,046*	37
Heart Rate	290	13,842	2,390,972	8,245
Weight	224	1945	2246	10
Blood Pressure	87	541	849	10

*Sleep and steps are a sum of all measurements recorded in a day.

Supplementary Table 2. Summary of surveys, frequency, timing and content

Short Intake	Health History	Weekly	Outcome
**1 time when join study**	**1 time when join study**	**Every week**	**4 weeks after due date or if participant indicates birth outcome in weekly survey**
**Date of birth**	No. of prior live births	Still pregnant? (Yes, No-pregnancy ended, No-miscarriage or stillbirth, date of loss (if birth, go to outcome survey questions)	Date of birth
**Race/ethnicity**	No. of prior miscarriages	Weekly weight (date, who took measurement)	Weight and length of baby(ies)
**Zip code**	Have you seen a health care provider since becoming pregnant?	Weekly blood pressure (date/time, who took measurement)	Sex of baby(ies)
**Due date**	Have you had an ultrasound during this pregnancy?	Weekly pulse rate	Labor induced
**Height**	How many babies are you carrying?	Weekly vomiting, nausea, lower abdominal cramping, none (how often)	Epidural
**Pre-pregnancy weight**	Prenatal vitamins	New medications	Type of delivery
	Medications (prescription, over the counter, or supplements)	Prenatal visit this week?	Place of birth (home, hospital, non-hospital birthing center, other)
	Vaccines	If yes, weekly vaccinations	
	Currently treated for anxiety or depression?	If yes, weekly pregnancy diagnoses	
	Pre-pregnancy conditions		
	Type of prenatal care provider		

Supplementary Figure 1. Unique views of the “read more” or detailed version of each eConsent topic. March 16, -December 17, 2017.

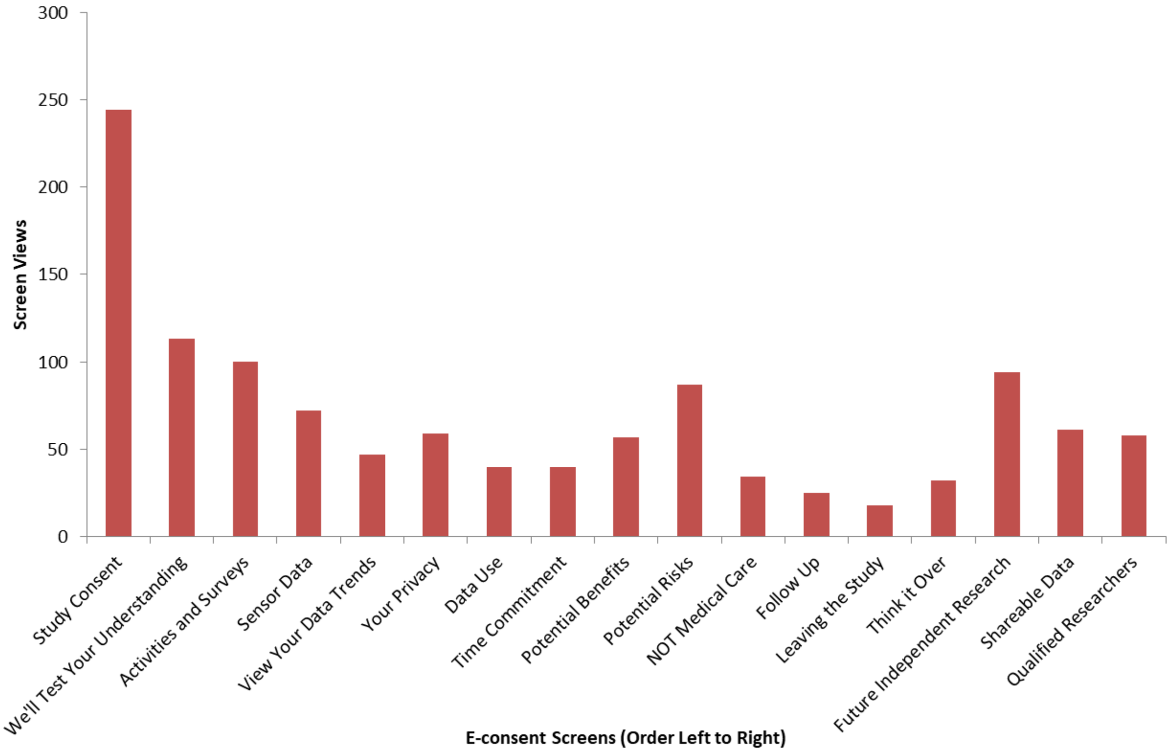

Supplementary Figure 2. Frequency count of participants by state. March 16, 2017- December 17, 2017

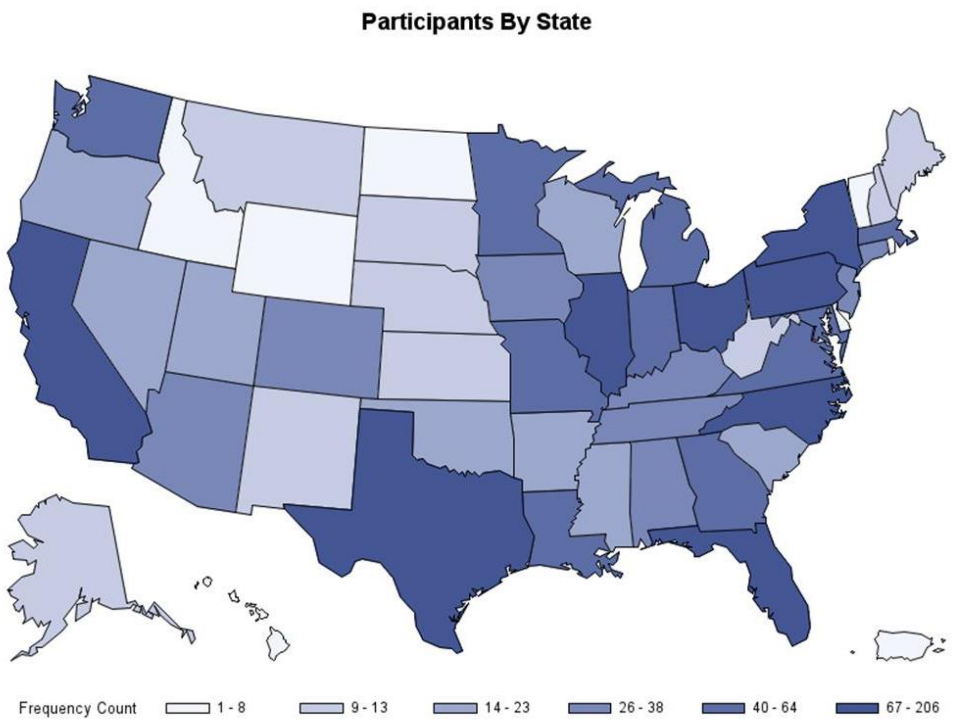

Supplementary Figure 3. Top: age distribution. Bottom: Body Mass Index (BMI) distribution. (blue) underweight (yellow) normal weight (green) overweight, and (red) obese.

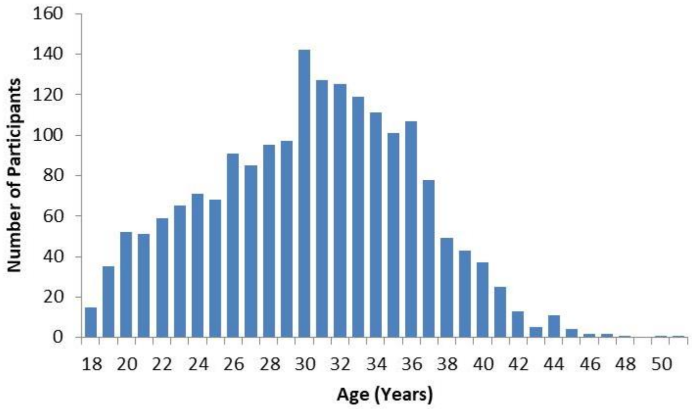

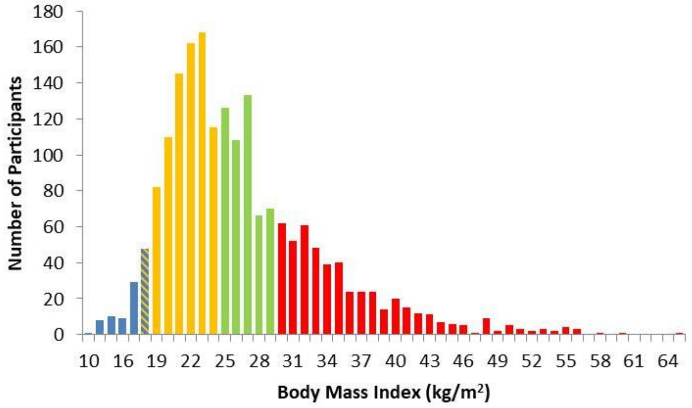

Supplementary Figure 4. Heatmap showing the number of self-reported weight measurements (from the weekly surveys) grouped by week of pregnancy when a participant joined the study. Also shown is the number out of outcome surveys filled out by week joined. Note: This is a data snapshot: some of these participants were still pregnant when the data was collected for this figure and therefore haven't had the opportunity to fill out all surveys. Data is only for singleton pregnancies.

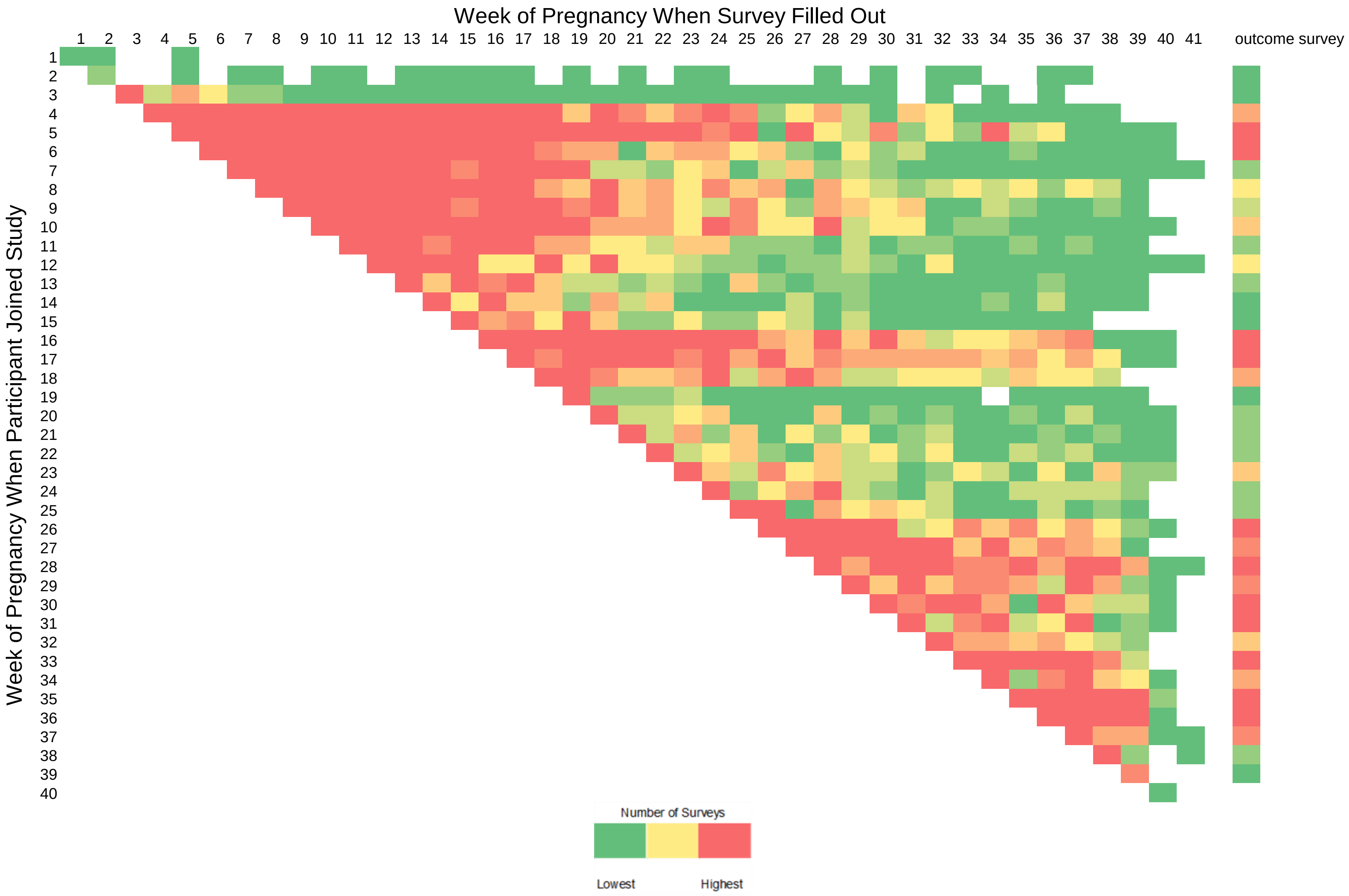

Supplementary Figure 5: Examples of Carousel Cards

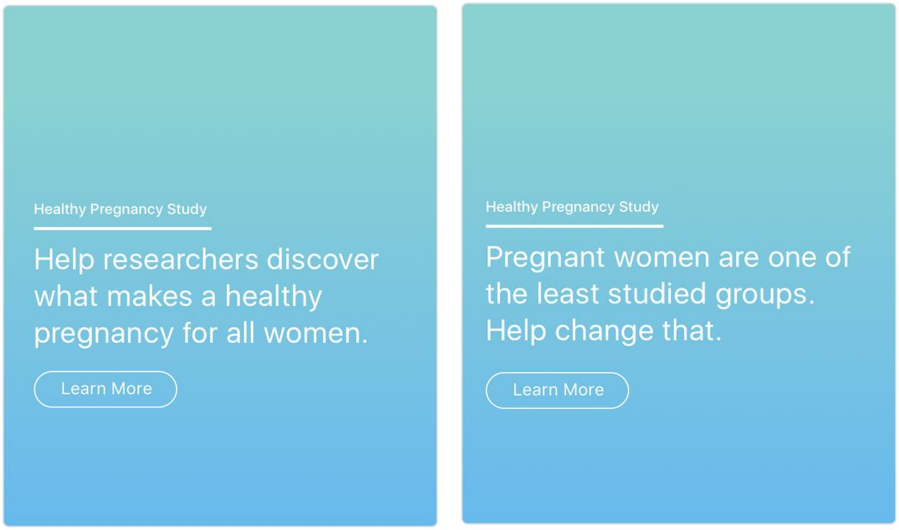

Supplement: Supplementary file 1 — Supplementary Information [file 41746_2018_52_MOESM1_ESM.pdf]
